# Families as Valued Contributors to LTC Residents' Quality of Life: Policy Perspectives

**DOI:** 10.1093/geroni/igab046.1426

**Published:** 2021-12-17

**Authors:** Janice Keefe

**Affiliations:** Mount Saint Vincent University, Halifax, Nova Scotia, Canada

## Abstract

Family members are essential contributors to Q0L of LTC residents. This paper analyzes how the system views family’s role in residents’ QoL and enables or inhibits family involvement. Our analysis of 21 policies that regulate LTC in four Canadian Provinces reveal differences in their portrayal of residents’ families. In many policies, family roles are characterized procedurally (task-oriented) or relationally (interactive) by policy type. Operational standards (regulatory policies) linked to licensing employ more formal terminology, while LTC program guidelines use facilitative language to engage families and build relationships through voluntary means. Specific examples of orientation and admission procedures, care protocols including use of restraints, right to live at risk, and end-of-life care are presented to reveal inter-provincial variations. We argue there are opportunities to further engage families within the current regulatory framework.

